# Progress in Surgery

**Published:** 1899-08-12

**Authors:** 


### Progress in Surgery.

### TUMOURS.

(*Continued from page* 317.)

**What can Surgery do for Cancer?**—This subject is discussed by Cheyne.[4] Cancer of the skin is mentioned as being the most favourable form of cancer. On the average it is slow-growing and often remains limited to the skin a long time, and even when the lymphatic glands are affected the lymphatic vessels running between the skin and the glands are seldom diseased. The best results are seen in epithelioma of the extremities, especially after amputation. Volkmann gives the cures thus effected as 51 per cent., and the recurrences, which are essentially glandular, and not in the stump, as 35 per cent. In rodent ulcer the results are particularly good. Sulphuric acid paste is recommended where free removal cannot be practised. Cancer of the lip does not yield extremely favourable results, probably from the fact that the muscle is intimately connected with the skin, and cancer cells are driven along them to glands at an early period. In operating, a wide wedge should be removed with the apex prolonged to the edge of the jaw. To thoroughly clear out the submaxillary glands a curved incision is recommended, starting in the middle line at the hyoid, running up to the jaw, and along it nearly to the angle, and downwards over the vessels. The fat, fascia, salivary gland, and lymphatic glands are then dissected off the muscles *en masse.* If these glands are large the upper part of the anterior triangle should also be cleared. Cancer of the tongue is one of the worst forms. Septic troubles after operation are to be guarded against by cleansing the mouth, injecting anti-streptococcic serum, application of chloride of zinc to the wound, and stitching up as much of the wound in the mouth as possible. The mortality ought not to exceed 5 to 10 per cent. The glands may be removed first, and the tongue a week later, thus saving septic complications spreading to the neck. If little or no glandular enlargement exists the tongue may be removed first. The clearing of the neck should be very thorough, for the cicatricial tissue left will prevent another removal of glands being successful. Operation is contra-indicated if the artery is affected, for ligature of the carotid is almost certain to be followed by hemiplegia and death. The vein may be removed. The vagus has several times been removed with impunity. Where radical cure is not possible the tongue may sometimes be removed with advantage. If that cannot be done, such operations as gastrostomy or tracheotomy are not recommended. In cancer of the throat the amenability to relief depends upon the situation and extent of the disease, the favourable cases being those where the disease affects the upper parts of the tract—the soft palate, the tonsil alone, or one or other faucial pillar. The worst forms are those where the growth is not limited to the pharynx, but has spread on to the orifice of the larynx. A third to a half of the circumference of the pharynx may be removed without marked trouble from stricture, especially if the wound is closed as much as possible by vertical sutures. Cancer of the larynx may be intrinsic and uni-lateral, intrinsic and bi-lateral, or both intrinsic and extrinsic. For the first form, thyrotomy and removal of the affected cord yields excellent results; for the second class it is often best to remove half the larynx or thyroid cartilage; for the third class, complete excision of the larynx is the only operation that can offer any sort of radical cure, and glands have to be removed. This excision yields much better results when the trachea is brought out at the skin wound and sutured there, the wound in the pharynx being closed, and communication between pharynx and larynx shut off. In cancer of the œsophagus it is stated that Symond's tubes should always be tried before gastrostomy is performed. If the cancer be high up they will not usually be tolerated.

**Inoperable Cancer.**—Coley[4] discusses the treatment of this condition. The results of the treatment with extract of Chelidonium Majus (celandine) in the cases collected by Spivak are mentioned. It is a drug which has been used for centuries in Asia and the East Indies, and been supposed to exercise a destructive influence upon warts, corns, and cancers. Of 61 cases collected by Spivak 33 showed improvement, 27 did not. Spivak concludes that the drug undoubtedly has some influence on cancerous growths. No case has remained well long enough to be considered cured. The treatment of tumours by electric puncture appears to be sometimes successful. Three cases of well-advanced cancer have been reported as cured by C. W. Reading, of Philadelphia. One was well three years and another seven years after treatment. They were not, however, of the inoperable class, but the patients refused operation. Massey's cases of treatment by kataphoresis are alluded to. It is based on the supposition that a relatively infinitesimal part of the oxychloride of mercury acts lethally on the cancer cells without destroying the normal tissues. This is discharged from the positive pole which is coated with mercury. It is not of much use if glands are involved. Some of Massey's results are striking, *e.g.*, a recurrent sarcoma of the palate disappeared under six weeks' treatment, and remained well a year. Parenchymatous injections of alcohol are said to have cured 15 cases of cancer of the breast out of 18 in the practice of Hasse. A 30 per cent. solution of alcohol is used at first. Later the strength is increased. The injections are made into the periphery just outside the tumour, and are supposed to obliterate the afferent and efferent vessels. English and American surgeons have used the method without benefit. It is only suitable for cases in which glands are involved, and is more painful than the operation of removal, and tedious. Carbide of calcium is a remedy credited with good results by Etheridge. Ries[5] believes the therapeutic effect is due to the production of quicklime. It has been used in the uterus after curetting. It seems obviously dangerous to use such a caustic in that cavity. Lymph gland extract is believed to be beneficial in cancer by Snow, who thinks that lymph glands tend to destroy malignant protoplasms by virtue of their secretion. Orthoform and nasophen applied locally, singly or mixed, are recommended. The former is a strong local anæsthetic, acting for long periods, viz., twelve to twenty-four hours, and is non-poisonous. Coley's treatment by the mixed toxins has been in his own hands successful in 24 cases in 148, *i.e.*, 15 per cent. In six of these there was later recurrence, leaving 12 per cent. finally cured. Binnie writes on the ligation of vessels[6] for the purpose of producing atrophy of malignant tumours. This practice can only render real service when the growth is in a region which obtains nourishment from one, two, or four principal pedicles. Thus such ligation will fail for growths in face, mouth, and pharynx. It may sometimes succeed in organs like the kidney, spleen, or uterus. Tuffier's results in cases of cancer of the tongue and uterus are quoted. In the former pain and hæmorrhage stopped for some weeks, but the tumour never ceased growing. In one uterine case the success was remarkable. Bleeding diminished, the general health improved, and the patient was able to leave the hospital. The four uterine pedicles had been tied. M. Hartmann (Paris) has used the method with no good results in cancer, but in fibroids the tumour sometimes decreased in size and symptoms subsided. Tuffier caused a fibroid to disappear by this treatment.

**Oophorectomy for Cancer of the Breast.**—Stanley Boyd[7]

and Herman[8] have recently written on this subject. Boyd has now operated seven times. Full details are given of all these cases. In addition, five cases of other surgeons are tabulated, viz,, of Beatson, Cheyne, and Herman. Boyd did not give thyroid extract in any of his cases until it seemed clear that the effect of the öophorectomy (if any) was exhausted. In all his cases the operation produced no ill effect upon body or mind of the patients. Summing up his results, the operation was indubitably beneficial in two cases; probably so in two more; absent or very doubtful in the other three. In the group of failures are the only two cases in which the operation was done after the climacteric. The disease was very advanced in one, and the recurrence growing fast in the other of them. Of the two most successful cases one is now, two years after oöphorectomy, almost free from cancer, and in excellent general health. The other is dead. The cancer nodules had scarcely disappeared before they began to reappear. Boyd thinks he added about a year to her life. He asserts that no one who watched these cases could doubt the influence of oöphorectomy in inducing the disappearance of the cancer tissue. With these may be placed Beatson's first case, in which all cancer, which was recurrent and of rather bad type, disappeared in eight months after the operation, and the patient remained free until nearly three years had elapsed. It appears that all secondary growths in the same case are not equally affected by the operation. It may be that the effects are due to the removal of ovaries secreting harmful material, and that in some cancer cases the ovaries are not at fault and others they are, so accounting for the differences in the effect of their removal. In two of his oöphorectomy cases Boyd gave thyroid extract, but not immediately. In Beatson's, Cheyne's, and Herman's the extract was given while the effects of the oöphorectomy were being observed. Boyd considers his cases give little evidence that thyroid extract affects the disease. In three out of his five cases the ovaries had not been removed. Herman's first successful case was one of recurrence. and she is now, more than two years after the oöphorectomy and nine years after the first operation on the cancer, quite free from recurrence. The second case, a woman of 49, was operated upon twice at the Middlesex Hospital in 1895. In 1897 she had recurrence in the scar, and also a stony hard lump had developed in the remaining breast. In July, 1898, Herman operated, and immediately put her on thyroid extract, grs. v., three times daily. In March, 1899, nine months after oöphorectomy, she presented herself with the recurrent ulcer soundly healed, and the other breast healthy except for retracted nipple. Herman points out that of the four cases published in which oöphorectomy, together with immediate administration of thyroid extract, was the treatment adopted, in three the cancers have disappeared. Page and Bishop have published a case in which the thyroid extract alone effected a cure. The combined treatment appears, therefore to be much the most effectual.

[4] Practitioner, April, 1899. [5] Jour. of Amer. Med. Assoc., Nov., 1898. [6] Annals of Surgery, Feb., 1899. [7] Brit. Med. Jour., Feb., 1899. [8] Lancet, April, 1899.

